# Implementing a low-threshold audio-only telehealth model for medication-assisted treatment of opioid use disorder at a community-based non-profit organization in Washington, D.C.

**DOI:** 10.1186/s12954-021-00578-1

**Published:** 2021-12-09

**Authors:** Ellis Jaewon Yeo, Hannah Kralles, David Sternberg, Dana McCullough, Ajetha Nadanasabesan, Richard Mayo, Hana Akselrod, Jillian Catalanotti

**Affiliations:** 1 HIPS Clinic, 906 H Street, NE, Washington, DC 20002 USA; 2 Washington AIDS Partnership, 1701 Rhode Island Ave NW, Suite 03-108, Washington, DC 20036 USA; 3 Department of Medicine, The George Washington University, 2150 Pennsylvania Avenue, Washington, DC 20037 USA

**Keywords:** Harm reduction, Opioid use disorder, Medication-assisted treatment, Buprenorphine, COVID-19, Telehealth

## Abstract

**Background:**

The COVID-19 pandemic has had especially devastating effects on people who use drugs. Due to pandemic protocols in the USA, medication-assisted treatment (MAT) regulations became more flexible, permitting our community-based nonprofit organization to transition its low-threshold MAT clinic to an audio-only telehealth model of care in 2020. Lessons learned have the potential to improve MAT delivery to people with OUD.

**Case presentation:**

This case study describes our transition from a low-threshold community-based in-person MAT clinic to an audio-only telehealth model. We extracted data from electronic health records to describe patient characteristics and to calculate treatment retention rates. Patients were predominantly male (74.4%) and black (90.6%). The mean age was 53 years old with more than half of the clients aged 55 or older. Less than half (42.3%) of the patients lived in stable housing. Patients commonly had self-reported comorbid conditions such as hypertension (35.4%), hepatitis C (23.5%), diabetes (11.9%), human immunodeficiency virus (HIV) (7.2%). A majority of patients (68.6%) reported engagement with behavioral health care. We measure the success of our intervention relative to published retention rates, both overall as well as for in-person and telehealth care. In-person retention rates at 90- and 180-days were substantially higher than telehealth retention rates (93.9% vs 68.4% and 91.5% vs 51.9%, respectively).

**Conclusions:**

Low-threshold medication-assisted treatment in the care of people with opioid use disorder is essential to increasing treatment access and continuity. We found that an audio-only telehealth model was viable. Although we had decreased retention rates following the transition to an audio-only telehealth model, our rates remained excellent compared to published values for in-person MAT care. We call for advocacy and regulations to support continued use of telehealth services throughout and beyond the COVID-19 pandemic.

**Supplementary Information:**

The online version contains supplementary material available at 10.1186/s12954-021-00578-1.

## Background

The COVID-19 pandemic has caused especially devastating effects on people who use drugs (PWUD), worsening isolation as a result of social distancing and increasing existing barriers to accessing treatment. In 2020, opioid-related deaths in Washington, D.C. hit an all-time high of 408 deaths—a 45% increase from 281 opioid related deaths in 2019 [1]. Further exacerbating disproportionate effects of the pandemic, many addiction treatment centers and harm reduction organizations reduced hours and services for PWUD in response to social distancing recommendations and regulations [2]. These interruptions in treatment and services placed already marginalized individuals at an increased risk of death and other drug-related harms [2].

Medication-assisted treatment (MAT) for opioid use disorder (OUD), such as buprenorphine-naloxone, decreases opioid use and fatal overdoses by reducing opioid withdrawal and cravings [3]. Strict federal and state regulations in the USA have historically created barriers to providing MAT, and the inflexibility of traditional care models prevents many PWUDs from initiating or remaining engaged in MAT [4]. In response to the COVID-19 pandemic, many restrictions were lifted, including the requirement for an in-person visit. On March 31, 2020, the Substance Abuse Mental Health Services Administration, the US Drug Enforcement Administration, and the US Department of Health and Human Services issued a statement allowing use of audio-only telehealth encounters for buprenorphine-naloxone induction without requiring an in-person evaluation or video interface [5]. This granted providers the option to transition to audio-only telehealth for both induction and follow-up MAT appointments throughout the pandemic.

An urban, community-based non-profit organization located in Washington D.C., offering non-judgmental harm reduction services and low-threshold MAT to people with OUD, transitioned to an audio-only telehealth MAT model in March 2020. Although traditional health care centers, including local hospitals and FQHCs, were overwhelmed due to clinical demands of the COVID-19 pandemic, this clinic continued to provide care to people with OUD and was notably the only MAT clinic in the Washington, D.C. metropolitan area to induct new patients from the local start of the pandemic in March 2020 until December 2020, initiating 111 people on buprenorphine-naloxone via telehealth during that time.

This transition to telehealth provided an opportunity to study the impact of a flexible, low-threshold model of audio-only telehealth care intended to increase treatment access for people with OUD [4]. Previous research shows that low-threshold treatment is associated with better retention overall, demonstrating the importance of ongoing consideration of this model beyond the pandemic [6]. This case study outlines the organizational telehealth adaptations that enabled virtual counseling, peer support, therapy groups, and clinical care during COVID-19 in this community-based MAT program. Lessons learned have the potential to improve MAT delivery to people with OUD.

## Case presentation

### Low-threshold MAT model

The community-based harm reduction program originally provided syringe exchange and advocacy for those engaged in sex work. In 2018, it launched a MAT clinical services program to fill gaps left by local federally qualified health centers (FQHCs). The patients served are marginalized by structural inequities beyond their opioid use disorder: low socioeconomic status, homelessness, previous incarceration, and chronic health conditions.

Our low-threshold MAT care model has certain aspects that differentiate it from other MAT clinics. Since opening the clinic in January 2018, we have refined our practices to promote low-threshold MAT induction and treatment continuation that is patient-centric. The staff is made up of a clinic coordinator, medical providers, and community health workers. The majority of our staff share lived experiences with clients, including recovery from substance use, and come from similar communities as the clients. Both factors allow us to build trust with the community and stand at the forefront of nonjudgmental harm reduction, advocacy, and community engagement.

We perform true team-based care; at intake, community health workers (CHWs) take past medical, psychosocial and substance use histories (see Additional file 1) and educate patients about buprenorphine-naloxone for MAT. Afterward, healthcare providers located on-site meet with patients to rule out medical contraindications and provide initial prescriptions, typically for one week of daily buprenorphine-naloxone 8-2 mg twice a day for 16 mg/day. At the first return visit, we use supervised in-clinic induction with a one-time buprenorphine-naloxone 8-2 mg dose when the patient has reached moderate withdrawal. We have had no episodes of precipitated withdrawal. During follow-up appointments, providers titrate MAT dosing to 24 or 32 mg/day, depending on the patient’s needs. CHWs inquire about social determinants impacting health and offer referrals to relevant services both internal and external to our organization, including substance use counseling, housing and employment assistance, identification and record procurement, sexual health and infectious disease services, and primary care or mental health providers.

To remain patient-centric, we do not perform phlebotomy at any time. We perform urine pregnancy testing of reproductive age women prior to therapy initiation, and periodic urine testing for buprenorphine- and its metabolite, norbuprenorphine, throughout the course of care, but we do not routinely perform urine drug screens (UDS) for other substances. No pregnancies have been reported. Our organization also partners with a local community-based pharmacy that delivers medication, intranasal naloxone, and harm reduction kits to our patients. Patients are encouraged, but not required, to use this pharmacy, whose employees are knowledgeable about the community and work in direct communication with the MAT clinic team. To decrease barriers further, we coordinate transportation to and from any location relevant to the patient’s care, at no cost to the patient using Uber Health © services (Uber Technologies, Inc. California, USA).

Finally, clients are never penalized for missing an appointment. Our flexible attendance policy allows clients to miss several appointments and resume care, even after periods of longer discontinuation, prioritizing the reduction of drug-related harms over abstinence. The clinic does not discharge patients with ongoing drug use and considers any reduction in opioid use to be a positive outcome.

### Transition to telehealth

As a result of the COVID-19 pandemic, our MAT clinic transitioned to an audio-only telehealth MAT model starting March 13, 2020. Existing patients were notified of the transition via phone calls. New patients were recruited through outreach at local homeless shelters, parks, and laundromats during which business cards with the clinic coordinator’s phone number were distributed. During the initial telephone visit, which included the same team-based components as our prior in-person visits, providers sent same-day prescriptions to our pharmacy partner and provided instructions for at-home induction: after at least 12 h of abstinence from opioids, and once the patient entered moderate withdrawal, they would take an induction dose of buprenorphine-naloxone 8-2 mg sublingually, repeating this dose after approximately 12 h if needed. Follow-up telephone visits include the same team-based care components as they did when in person. Community health workers first call patients to complete the social history and offer referrals to services, and then connect them to the medical provider.

Additionally, we substantially reduced urine drug testing throughout treatment, only performing UDS if required by a patient’s health insurance. This decision was initially made due to the need to limit face-to-face interactions during the COVID-19 pandemic, but is supported by the philosophy that diverted buprenorphine-naloxone is often used therapeutically by others in the community and may, therefore, present minimal harm and possible community-level benefit [7–9].

## Data

In January 2021, we retrospectively extracted demographic and clinical information from electronic medical records for patients enrolled in the MAT clinic during the entirety of its history (January 2018–December 2020). We used descriptive statistics to analyze the overall data for our in-person low-threshold MAT clinic and for telephone-based MAT clinic (Table 1).

**Table 1:** Overall demographic and clinical characteristics of patients

Baseline characteristics	Total *n* (%)
Age (mean years ± SD)	52.8 ± 11.4
Male	206 (74.4)
*Race/ethnicity*	
Black	183 (90.6)
White	18 (8.9)
Hispanic	1 (0)
*Current housing*	
Unhoused	73 (26.6)
Living in own place	116 (42.3)
Living with others	78 (28.5)
Transitional	7 (2.6)
*Employment*	
Full time	17 (6.3)
Part time	32 (11.8)
Not working	184 (67.6)
Looking for work	39 (14.3)
*Route of drug use*	
Injection	47 (17.3)
Snort	154 (56.8)
Both	70 (25.8)
Ever overdosed	94 (33.8)
*Substance use history*	
Prescribed suboxone	69 (24.9)
Prescribed methadone	55 (19.9)
Prescribed suboxone and methadone	24 (8.7)
“Street” suboxone	56 (20.2)
“Street” methadone	5 (1.8)
“Street” suboxone and methadone	7 (2.5)
Crack/cocaine	78 (28.3)
Marijuana	56 (20.3)
Meth	11 (4.0)
PCP	45 (16.3)
Benzos	23 (8.3)
Alcohol	23 (8.3)
Tobacco	174 (63.0)
*Chronic health conditions*	
HTN	98 (35.4)
Diabetes	33 (11.9)
HIV positive	20 (7.2)
On HIV treatment	15 (5.4)
HCV positive	65 (23.5)
Already treated	50 (18.1)
Treated in our ID Clinic	19 (6.9)
*Mental health conditions*	
PTSD	39 (14.1)
Schizophrenia	30 (10.8)
Bipolar	59 (21.3)
Depression	112 (40.4)
Anxiety	62 (22.7)
*Engaged in behavioral health care*	
Yes	190 (68.6)

### Patient characteristics

Overall, between January 2018 and December 2020, 277 patients were enrolled in MAT. Demographic data were self-reported at induction. Patients were predominantly male (74.4%) and black (90.6%). The mean age was 53 years with more than half of the clients aged 55 or older. Less than half (42.3%) of the patients lived in stable housing, 26.6% were unstably housed (lived in a shelter or otherwise experienced homelessness), 28.5% were temporarily living with family and friends, and 2.6% were in transitional housing. Most patients (67.6%) were not currently working, while 18.1% of patients had either part-time or full-time employment.

The age at which patients started using drugs ranged from 9 to 64 years old, with a mean age of 27 (SD 12.5). When asked about their route of drug use, 56.8% of patients reported snorting, 17.3% injecting, and 25.8% reported both snorting and injecting. 33.8% of patients reported a history of drug-related overdose. Two-thirds of the patients (*n* = 184) reported a prior history of Medication for Opioid Use Disorder (MOUD), including buprenorphine-naloxone and/or methadone. Of those who had prior history of MOUD, 36 (19.6%) had used solely MOUD obtained on the street, 116 (63.0%) had used prescribed MOUD, and 32 (17.4%) had used both street-obtained and prescribed. For both street-obtained and prescribed MOUD, buprenorphine-naloxone was more common than methadone. A large majority (87.4%) reported polysubstance use; substances used included tobacco (63.0%), crack/cocaine (28.3%), marijuana (20.3%), and PCP (16.3%).

Patients commonly had self-reported comorbid conditions such as hypertension (35.4%), diabetes (11.9%) and human immunodeficiency virus (HIV) (7.2%). Sixty-five patients (23.5%) had been diagnosed with hepatitis C, of whom 50 (76.9%) had been treated. Nineteen of the treated patients (38.0%) had received hepatitis C treatment in our on-site infectious disease clinic. One hundred ninety patients (68.6%) reported engagement with behavioral health care. Many patients reported mental health diagnoses, with 40.4% reporting depression, 22.7% anxiety disorder, 21.3% bipolar disorder, 14.1% post-traumatic stress disorder and 10.8% schizophrenia.

### Retention rates

We calculated the overall retention rate as the percentage of patients whom we had inducted on buprenorphine-naloxone and remained in treatment with us as of December 2020. We also calculated the percentage of patients who stayed in the program for 90 or 180 days after their induction. We excluded patients who started treatment after October 1, 2020, from the 90-day retention rate calculation and those who started after July 1, 2020, from the 180-day retention rate calculation. We calculated a 365-day retention rate for patients inducted in person only, as telehealth inductions began less than a year prior to our analysis.

Overall, among 277 patients inducted on buprenorphine-naloxone, 192 (68.6%) were still in treatment with us as of December 2020 (Table 2). The most common reasons for discontinuation are listed in Table 2. Since transitioning to an audio-only telehealth model, 111 people were inducted, and 72 (65%) of them were still in treatment with us as of December 2020. In-person retention rates at 90- and 180-days were substantially higher than telehealth retention rates (93.9% vs 68.4% and 91.5% vs 51.9%, respectively) (Table 2).

**Table 2:** Retention rates as of December 2020 and reasons for discontinuation for patients inducted on buprenorphine-naloxone therapy (*n* = 277)

	Number (%)
*Overall*	277
Continued treatment as of December 2020	190 (68.6)
90-day retention	223 (84.5)
180-day retention	192 (79.0)
*In-person inductees*	166
90-day retention	156 (93.9)
180-day retention	151 (91.5)
365-day retention	110 (79.1)
*Telehealth inductees*	111
90-day retention	67 (68.4)
180-day retention	40 (51.9)
*Overall discontinued patients*	87 (31.4)
Reason unknown	53 (19.1)
Transferred MAT care elsewhere	23 (8.3)
Deceased	5 (1.8)

## Discussion and conclusions

Low-threshold approaches to MAT have been associated with better retention in care [6, 10]. This may be even more crucial for patients of racial minority backgrounds, for whom MAT retention has been shown to be lower, likely due to the impact of complex and interacting social determinants [11]. Even before the COVID-19 pandemic, telemedicine was considered a potential strategy to increase access to MAT for patients in hard-to-reach rural communities [4, 9, 12]. In this case study, we describe an urban low-threshold buprenorphine-naloxone MAT program for people with OUD that was successful in inducting and retaining patients in continued treatment with buprenorphine-naloxone using both in-person care delivery and using telehealth.

Retention rates for our intervention were comparable to or better than rates reported in the literature. A systematic review studying retention in MAT suggests widely varying rates: 19.0–94.1% at 90 days 3.0–88.0% at 180-days and 37.0–90.7% at 365 days [13]. One urban community health center reported a 90-day retention rate of 70.7%, and a harm reduction agency in New York City reported patient retention rates of 68%, 63% and 42% at the end of 3, 9 and 12 months, respectively [9, 12]. Lower retention rates have been reported among people experiencing homelessness (53%, 44%, and 26%, respectively) [10]. Long-term retention in buprenorphine-naloxone treatment has not been widely studied, though it is commonly accepted that retention rates are likely to decrease as the duration of follow-up increases [12].

Our program’s in-person retention rates (94.0%, 91.6%, 79.1% at 90, 180 and 365 days) are substantially higher than typical rates and highlight the success of our low-threshold model of care before the COVID-19 pandemic, especially when viewed in light of the fact that fewer than half of our patients had stable housing.

One study examining the changes MAT clinics adopted since the COVID-19 pandemic reported that almost two-thirds of clinics reported an unchanged or easier time retaining and engaging patients, perhaps due to protocol adaptations, including writing longer duration prescriptions of buprenorphine-naloxone and reducing the frequency of urine drug screenings [8, 9]. Recent studies have reported success with transitioning MAT to a telehealth model, although typically programs utilized video telehealth [14]. Due to the low rates of cell phone and computer ownership among our patient population, we used audio-only telehealth to improve our accessibility. Our lower telehealth compared to in-person retention rates could be explained by several factors. Our clinic expanded in 2020 after hiring a new buprenorphine-waivered provider, which may confound direct comparison before and after telehealth began. The pandemic may have created increased mental, financial or psychosocial stress for patients, potentially making it more challenging for them to continue care. Patients may not have felt as engaged or connected to providers whom they may never have seen in person. Additionally, some patients had trouble accessing phones on a consistent basis. In 2021, we received a grant for cell phone distribution that we hope will help increase telehealth retention rates. Overall, our results are promising despite the decrease in retention rates when using telehealth. Although there are currently no published audio-only telehealth retention rates for comparison, our telehealth retention rates fall on the higher end of published ranges for in-person treatment.

As we plan for both the re-emerging need for precautions with the rise of COVID variants and an eventual return to normalcy in a post-COVID era, it is important to consider the benefits of telehealth for expanding access to MAT. Currently fewer than one-quarter of opioid dependent US individuals receive addiction treatment [15]. The telehealth model not only protects the health of patients and staff, it can also ensure continuity and provide low-threshold care. While some people may benefit from the structure and support of an in-person MAT program, others may find it burdensome. Compared to video telehealth, audio-only telehealth may be even more accessible to patients from marginalized populations, who may lack access to computers, cell phones or wireless internet. Addiction specialists have called for the sustainable expansion of these new models of low-threshold care beyond the pandemic [4]. We advocate for provision of MAT care via a low-threshold hybrid telehealth/in-person MOUD treatment model that can balance structure and flexibility and be tailored to each individual patient’s needs.

### Strengths and limitations

As above, several external and organizational factors may confound our ability to directly compare retention rates before and after the COVID pandemic. Despite this, strengths of our study include the relatively high number of patients, 40% of whom were inducted via telehealth, and our history of providing low-threshold MAT for just over two years before transitioning to telehealth, which allowed us to improve our low-threshold, team-based model of care before venturing into a new medium of care delivery. Due to our extensive intake questionnaire and electronic medical record, our demographic information is fairly robust. Staff were available to assist patients in answering demographic questions, decreasing literacy or educational barriers to correct responses. That said, some patients did not complete all the questions. It is possible that omissions, while not common, may slightly skew results. We do not believe this would have occurred in a systematic way. Finally, the findings of single descriptive case studies cannot be generalized to other settings. Our intervention was performed within the existing structure of an urban non-profit community organization with a patient population that was one of the most vulnerable in our area. We hope that sharing this description of our intervention, along with accompanying data demonstrating its success, will provide insight into potential changes in MAT care as providers around the world prepare for the future.

## Conclusion

Prior to the COVID-19 pandemic, rigid state and federal regulations created numerous barriers for people seeking MOUD in the USA, which may have prevented people from beginning or continuing care. The COVID-19 pandemic exposed inadequacies in traditional care models and opened the door for a new, highly innovative MOUD care model that centers around patients’ unique needs or circumstances. An audio-only telehealth model of MAT not only satisfies pandemic-related needs for social distancing and limited in-person interactions, but also was highly effective in providing treatment access with excellent retention rates.

As new COVID-19 variants arise and challenges to vaccine distribution and uptake continue worldwide, deciding which innovations borne of the pandemic to permanently institute becomes increasingly relevant. We urge harm reduction organizations, substance use treatment professionals, policy makers, and relevant agencies to advocate for audio-only telehealth as a standard acceptable option for MOUD care. Employing an audio-only telehealth model in the treatment of OUD, supplemented by additional low-threshold practices, provides vulnerable communities with safe, effective, patient-centered care.

## Supplementary Information

**Additional file 1:** Social History Questionnaire at Induction.

## Data Availability

The datasets used and/or analyzed during the current study are available from the corresponding author on reasonable request.

## Abbreviations

PWUD: People who use drugs
MAT: Medication-assisted treatment
OUD: Opioid use disorder
FQHC: Federally qualified health center
CHW: Community health worker
UDS: Urine drug screening
MOUD: Medication for opioid use disorder
