# Supplementary material for: Implementing a low-threshold audio-only telehealth model for medication-assisted treatment of opioid use disorder at a community-based non-profit organization in Washington, D.C
Source: Harm Reduct J. 2021 Dec 9;18:127. doi: 10.1186/s12954-021-00578-1 (PMC8655329; doi:10.1186/s12954-021-00578-1)
Supplement: Supplementary file 1 — Additional file 1: Social History Questionnaire at Induction. [file 12954_2021_578_MOESM1_ESM.pdf]

Social History Questionnaire at Induction

1. How has drug use affected your life?
2. What age did you start using drugs?
3. How frequently do you use opioids?
4. Do you snort or inject or both?
5. Have you experienced an overdose?
6. Have you previously used buprenorphine or methadone?
7. If yes, was it prescribed by a doctor?
8. What other drugs do you use?
9. Do you use any alcohol, tobacco, or benzos?
10. Do you take any medications?
11. Do you have any chronic health conditions?
12. Do you know your HIV/ HCV status?
13. If positive for HCV or HIV, have you been treated or are you in treatment?
14. Do you have a primary care provider?
15. Do you have a mental health care provider?
16. Are you experiencing any legal issues?
17. Where are you currently living?
18. Do you have a photo ID card?
